# Date Seed: Rich Source of Antioxidant Phenolics Obtained by Hydrothermal Treatments

**DOI:** 10.3390/antiox11101914

**Published:** 2022-09-27

**Authors:** Abdessalem Mrabet, Ana Jiménez-Araujo, África Fernández-Prior, Alejandra Bermúdez-Oria, Juan Fernández-Bolaños, Marianne Sindic, Guillermo Rodríguez-Gutiérrez

**Affiliations:** 1 Instituto de la Grasa, Consejo Superior de Investigaciones Científicas (CSIC), Campus Universitario Pablo de Olavide, Edificio 46, Ctra. de Utrera, 41013 Seville, Spain; 2 Department of Food Technology, University of Liege—Gembloux Agro-Bio Tech, Passage des Déportés, 2, B-5030 Gembloux, Belgium

**Keywords:** phenolics, natural extracts, wastes, by-products, antioxidants, date seed, hydrothermal treatments

## Abstract

The growing interest in natural compounds is helping to improve the management of agro-industrial by-products such as the date seed as sources of such compounds. In this work, the application of a hydrothermal treatment at 160 and 180 °C for 60 min was studied to achieve the solubilization of its phenolic components and sugars in order to obtain biologically active extracts. The percentage of phenols and total sugars in the final extracts were very similar, at 45 and 25% for the 160 and 180 °C treatments, respectively. The treatment at a higher temperature allowed greater solubilization of other components. The antioxidant activity was measured as free-radical scavenging capacity. For the DPPH^•^ method, expressed as EC50, the results were 0.34 and 0.37 mg/L, the TEAC values for the ABTS^•^ method were 6.61 and 3.28 mg/g dried extract, and the values obtained by the ORAC method were 12.82 and 9.91 mmol Trolox/g dried extract, for 160 and 180 °C, respectively. All these values are higher than those of other plant extracts and extracts obtained using the whole date. Therefore, the date seed is a very important source of phenols, and through thermal and chromatographic processes, it is possible to obtain extracts with high antioxidant activity.

## 1. Introduction

The growing importance of natural products with biological properties which improve our health is based not only on improving what we eat by replacing synthetic components with natural ones, but also on promoting better use of the waste and effluents generated by the agri-food industry. In this sense, they are no longer called waste and the term by-product is increasingly being used, as they are being exploited more and more in other applications. This has an impact on both human and environmental health, which are closely related. This is the case of the by-products derived from dates, such as the less commercial secondary varieties and seeds. The secondary varieties have been studied to offer alternatives and improve their properties, such as obtaining pastes, antioxidant fibers, or phenolic extracts [1,2]. The date is one of the oldest crops, mainly developed in the Middle East and North Africa [3]. Dates are a rich nutritional source and a recognized source of biologically active components [4]. One of the main by-products of dates is the seed, which was initially used only for animal feed, although more and more studies are showing its true potential [5,6]. Thanks to these studies, its uses are being oriented towards nutritional purposes, such as its use in fiber powder, coffee, bread, or even cosmetics and functional ingredients [5].

Not many studies have been carried out to promote the utilization of date seeds, but those that exist clearly highlight the interest in their fat composition and phenol content. The extraction of oil is an important starting point for its industrialization [6] followed by the extraction of bioactive components such as phenols. In this regard, mainly acid phenols and flavonoids have been identified, which have been extracted with different solvents after a grinding phase, proving their antioxidant [5,7,8,9,10,11] and anti-inflammatory activities [12], among others.

The aim of the present work is to study the obtaining of an extract that is rich in phenolic components from date seeds in order to improve their utilization. For this purpose, the application of a hydrothermal treatment system already used with other agro-industrial by-products was applied [13] and served to improve their use at an industrial level. It has also been studied previously for the use of secondary varieties of date using the whole date, or on the seed for the extraction of its oil [6,14]. The application of this heat treatment followed by the use of chromatographic systems will make it possible to obtain an extract with antioxidant and other properties, both at the laboratory level for analysis and study and at the industrial level for possible marketing.

## 2. Materials and Methods

### 2.1. Sample Preparation

Date seeds were obtained from the Jerid area of southern Tunisia. The seeds were washed with water to remove the remaining pulp and then dried at room temperature and air-dried. The average humidity was 12.6%. They were then dried in an oven at 60 °C for one day. Finally, the seeds were stored at −20 °C until use.

### 2.2. Hydrothermal Treatments and Fractionation of the Liquid Fractions

The thermal treatments used are based on the use of direct steam in a closed reaction chamber. For this purpose, a 100 L capacity reactor with a lower steam inlet was used in a closed pressure system, where two treatments were carried out: the first one at 160 °C at 5.5 Bar and the second one at 180 °C at a pressure of 9.1 Bar, both for 60 min. Two-kilogram samples were used in each treatment. At the end of the reaction time, the reactor was cooled with water at room temperature through a heating jacket. The wet treated material was filtered by centrifugation at 4700× *g* (Comteifa, S.L., Barcelona, Spain) to separate the solids and liquids, and the samples were stored at −20 °C before fractionation and analysis.

**Figure 1:**
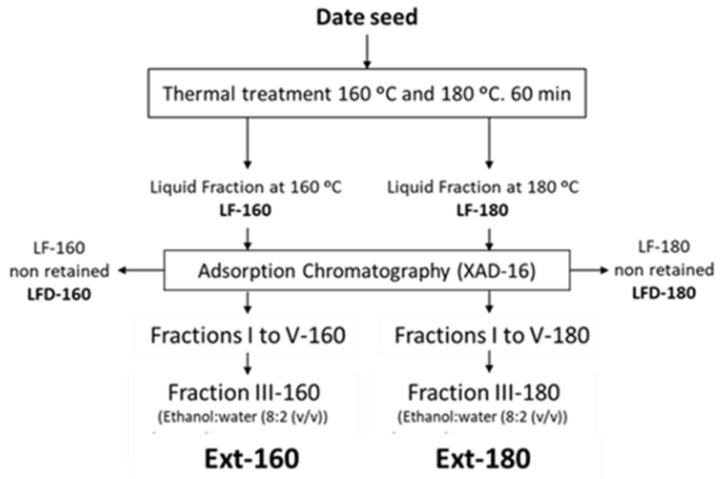
Treatment scheme for date seed samples and the main fractions studied.

The liquid fractions obtained after the two hydrothermal treatments (LF-160 and LF-180) (Figure 1) were fractionated through an adsorption column with XAD resin. The resin was pre-conditioned with pure ethanol and distilled water, using 10 times the resin bed volume in each case. Subsequently, 1 L of the liquid fraction obtained at 160 °C and 1 L of the liquid fraction obtained at 180 °C were fractionated using 800 mL of XAD-16 resin. Elution was performed with two 0.5 L fractions of distilled water (fractions I and II), followed by two 0.5 L fractions of an ethanol:water mixture (8:2 (*v/v*)) (fractions III and IV), and finally with 0.5 L of 100% ethanol (fraction V). Each of the fractions was stored at −20 °C until analysis. Fraction III of each treatment was chosen as the final extract (Ext-160 and Ext-180).

### 2.3. Determination of Total Sugars

The determination of total sugars was carried out using the anthrone–sulfuric acid method with a colorimetric measurement at 520 nm [15] in an iMark microplate absorbance reader (Bio-Rad, Hercules, CA, USA). Results are expressed as milligrams of glucose per liter, as the calibration line is made using different glucose concentrations.

### 2.4. Determination of Total Phenols

The amount of total phenols was determined by the Folin–Ciocalteu method [16]. It is a spectrophotometric method in which absorbance is measured at 760 nm. The results were expressed as milligrams of gallic acid equivalent per liter of each liquid fraction (mg GAE/L).

### 2.5. Analysis of Phenols by HPLC-DAD

Individual phenolics were quantified by high-performance liquid chromatography (HPLC). The equipment used was Hewlett-Packard 1100 liquid chromatography system with a diode array detector (DAD) using the following wavelengths for quantification: 254, 280, and 340 nm. A C-18 column was used (Teknokroma Mediterranea Sea 18, 250 mm × 4.6 mm, i.d. 5 μm), and the mobile phase was 0.01% trichloroacetic acid in water and acetonitrile. The gradient used for a total run time of 55 min was as follows: 95% A initially, 75% A in 30 min, 50% A in 45 min, 0% A in 47 min, 75% A in 95 min, and 95% A in 52 min until completion of the run. Quantification was performed by integration of the peaks at different wavelengths, depending on each compound, with respect to the calibration curves made using external standards.

### 2.6. Chemicals

Hydroxymethylfurfural (HMF), furfural, vanillic acid, protocatechuic acid, p-hydroxybenzoic acid, syringic acid, quercetin, and luteolin were obtained from Sigma-Aldrich (Deisenhofer, Germany). Ethanol was obtained from Fluka (Buchs, Switzerland). HPLC-grade acetonitrile was purchased from Merck (Darmstadt, Germany), and ultrapure water was obtained using a Milli-Q water system (Millipore, Milford, MA, USA). Methanol was obtained from Romil Ltd. (Waterbeach, UK).

### 2.7. Determination of the Antiradical Activity

#### 2.7.1. Antiradical Activity: 2,2-diphenyl-1-picrylhydrazyl (DPPH^•^)

The antioxidant activity of each extract obtained from both thermal treatments after fractionation in absorbent resin was determined as the free-radical scavenging capacity using the DPPH^•^ method described in a previous study [17]. The radical scavenging capacity of each antioxidant was expressed as EC50 (effective concentration, mg/L), as calculated from a calibration curve using linear regression for each final extract.

#### 2.7.2. Antiradical Activity: 2,2′-azino-bis(3-ethylbenzothiazoline-6-sulphonic acid) (ABTS^•^)

The ABTS^•^ assay was performed following the ABTS^•^ method [16]. The results were expressed in terms of the Trolox equivalent (TE) antioxidant capacity (TEAC) in mmol TE/g of dry extract.

#### 2.7.3. Oxygen Radical Absorbance Capacity (ORAC)

Determination of the oxygen radical absorbance capacity was carried out using the ORAC method [18] with modifications. Fluorescence was measured using excitation and emission wavelengths of 485 nm and 538 nm, respectively, with a measurement interval of 5 min for a total reading time of 90 min, using a fluorescent plate reader Fluoroskan Ascent FL (Thermo Scientific, Waltham, MA, USA).

The area under the curve was determined for all the samples, according to the following expression:

A = (0.5 + f5/f0 + f10/f0 + … + f90/f0) × int

where A is the area under the curve, f0 is the fluorescence value at time zero, fi is the value of the fluorescence at different times, and int is the time interval at which each measurement was made.

A calibration line was obtained for the area values of the different Trolox solutions. The final result was expressed as millimoles of Trolox equivalents (TE) per kilogram of fresh weight.

### 2.8. Statistical Analysis

Results were expressed as mean values ± standard deviations. STATGRAPHICS Centurion 18 software was used for statistical analysis. Comparisons amongst samples were made using one-way analysis of variance (ANOVA) and the LSD method. A *p*-value of 0.05 was considered significant.

## 3. Results

### 3.1. Hydrothermal Treatments and Fractionation: Phenolic Extract

**Figure 2:**
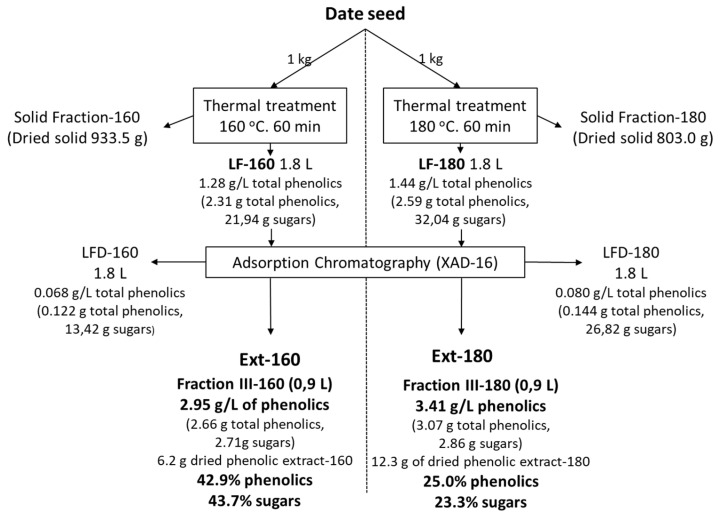
Balance of matter of the thermal treatment extraction and the chromatographic purification, based on one kilogram of seeds.

The thermal treatments were carried out at 160 and 180 °C. These conditions were chosen based on previous results found for secondary varieties of dates [2]. In both treatments, two kilograms of seeds were used, and a treatment with direct steam was applied. Finally, the samples were filtered to separate the liquid and solid fractions. The present work focused on the liquid fractions. Figure 2 shows the balance of matter based on one kilogram of seeds. With regard to the solid fraction, it should be noted that the treatment made it possible to make better use of it, since it facilitates its grinding and the extraction of the fat, as shown in a previous study [6]. The same amount of liquid fraction was obtained in both treatments, with a solubilization of 6.6% for the 160 °C treatment and much higher in the case of the 180 °C treatment, at 19.7%. This difference is not so clearly reflected in the content of solubilized phenols but in the content of solubilized sugars. Both extracts (Ext-160 and Ext-180) showed high phenolic percentages, 43% for the 160 °C treatment and 25% for the 180 °C treatment, somewhat lower for the latter, as more of the other components were solubilized. It is also important to note that the percentage of sugars present in the extracts is very similar to that of phenols; in the case of the 160 °C treatment, it represents almost the totality of the extracts.

Table 1 shows the amounts of phenols and sugars solubilized after thermal treatment and also the fraction not retained on the XAD chromatographic column and one of the five fractions, which corresponds to the one made with ethanol:water (8:2 (*v/v*)), which was chosen because it contained the highest concentration of phenols. Treatment at 180 °C slightly improves the solubilization of sugars and phenols. After the application of a chromatographic system, the amount of extract in the liquid fraction is higher after the higher temperature treatment, obtaining up to 3.41 g/L of total phenols, although the solubilization of a greater amount of other components, practically double (12.3 g), means that the purity of these phenols is lower in the Ext-180, almost half, at 25% compared to 43% for the 180 °C and 160 °C treatments, respectively, with regard to dry matter.

The table shows the data relating to the purification phase of the two liquid phases through the XAD-type adsorption chromatographic columns. After charging LF, the phenols retained in the column were eluted first with water (fractions I and II) to promote the elution of the sugars and then with a mixture of ethanol and water (8:2 (*v/v*)) (fractions III and IV) and finally with pure ethanol (fraction V). In both cases, it is observed that most of the phenols are eluted with the mixture of ethanol and water, mainly in the first faction obtained with this mixture. It is also observed that the sugars are eluted throughout the whole process, being more concentrated in the first elution fraction with water and also in the first fraction of the elution with the ethanol–water mixture. This fact may indicate that the type of sugars that are eluted with a high percentage of ethanol may contain phenolic residues and/or acid sugar or pectin, with or without phenolics, which causes them to be absorbed in the column in the same way as phenols. The union of sugars to phenols is favored by the application of this type of hydrothermal treatment, as has already been demonstrated with other substrates [19].

### 3.2. Characterization and Antioxidant Determination of Phenolic Extracts

**Figure 3:**
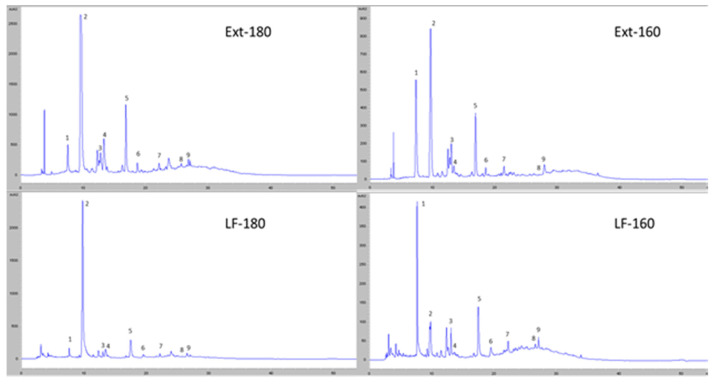
Chromatographic profile obtained by HPLC-DAD at 280 nm of the liquid fractions obtained from the hydrothermally treated date seeds at 160 °C (LF-160) and 180 °C (LF-180), their phenolic extracts (Ext-160 and Ext-180), and the main compounds detected: 1: gallic acid, 2: hydroxymethylfurfural, 3: protocatechuic acid, 4: furfural, 5: vanillic acid, 6: p-hydroxybenzoic acid, 7: syringic acid, 8: luteolin, and 9: quercetin.

Figure 3 shows the chromatographic profiles at 280 nm for the two liquid fractions obtained after hydrothermal treatment and for the two phenolic extracts from these fractions. It can be seen how the treatment at 180 °C increases the amount of degradation products, such as furfurans, while maintaining a very similar phenol profile to that of the fractions obtained at lower temperatures.

Finally, the fractions obtained in the elution with the first methanol–water mixture (III-160 and III-180) were considered to be of greatest interest due to their high phenol concentration. These fractions were characterized in comparison with the liquid fractions obtained directly after the heat treatments, as shown in Table 2. The antioxidant activity of these extracts was also determined through the DPPH^•^, ABTS^•^, and ORAC antiradical capacity assays, also shown in Table 2.

## 4. Discussion

In terms of phenolic acids, significant differences were observed between the treatments, with solubilization being greater at 180 °C, with the exception of gallic acid. In the extracts, the concentration of all phenolic acids is higher, and between the two temperatures, the highest quantity is still found at 180. The same happens with the flavonoids, with the particularity of quercetin, which increases fundamentally in the purification phase of the higher temperature fraction. There is no significant difference in the contents of phenols and total sugars in the liquid fractions after the two heat treatments, but there is a significant difference in the extracts obtained. The contents of hydroxymethylfurfural and furfural, which are products of the thermal degradation of sugars, more specifically of hexoses and pentoses, respectively, have also been determined. The content of these compounds increases drastically with increasing temperatures and they become concentrated in the extracts. There is great controversy regarding the toxicity of these degradation compounds, evidenced by in vitro tests but not in vivo tests [20]. Despite this, the concentration in which it is found in the extracts and the amount of extract that would be used in food for the prevention of oxidation would be very low, being well below the 30–40 mg per person that is the estimated normal consumption as they are present in many foods.

The main objective of this work was to evaluate whether a hydrothermal treatment helps date seed valorization, improving the solubilization of the main compounds, such as phenols and sugars. Previous work has already verified that this type of treatment allows better accessibility to the rest of the solid components such as fat [6]. Bijami et al. [5] studied how the mineral and phenol compositions varied with seed development. The maximum concentrations of acid phenols found by these authors were similar in the case of protocatechuic acid and lower in the case of gallic acid, 45% lower than that achieved after hydrothermal treatments. In the case of quercetin, which was not detected by these authors, its concentration increased substantially thanks to the pretreatment. Other authors improved the extraction by increasing the specific surface area, obtaining a seed powder from which they carried out extractions with water, obtaining an extract with 2–4.7 mg/100 g [9], or using hydroalcoholic mixtures as extracting agents, reaching levels of total phenols of between 11 and 71 mg/100 g [8]. These values are still far below the total phenolic values obtained in the present work, which are above 245 mg/100 g. Liu et al. [10] improved phenol extraction after grinding date seeds using a supercritical fluid extraction of up to 441.57 mg of total phenolics/100 g fresh weight. Nevertheless, other authors quantify higher amounts of total phenols and flavonoids, improving not only the specific surface area by grinding below 1.5 mm, but also by making sequential extractions, up to four times, with solvents such as water, ethanol, methanol, acetone, or their mixtures [7,11], where the maximum amounts of total phenols and flavonoids were between 11 and 3 g/100 g of date seed, respectively, although the total phenolic acid content did not exceed 194 mg/100 g. While some of these values are much higher than those obtained in this work, applying a thermal treatment with steam without grinding the seed seems a more easily scalable solution at the industrial level, and more so considering a subsequent concentration step through adsorption resins. This makes it easier and cheaper to obtain an extract that is rich in phenols and has high antioxidant activity. In these treatments, it is necessary to take into account the high costs of grinding a material such as date seed, which raises the price of the extracts obtained, and more so if techniques that imply high investment costs are added. In this sense, the use of thermal treatments has already been scaled [13], and economic studies have shown its feasibility with positive balances where not only the phenolic extract but also the rest of the components such as sugars and fiber for feed [14] or even for energy production [21] must be taken into account.

Regarding the antioxidant capacity determined by different methods for the extracts, it should be noted that they are all higher for the extracts obtained after treatment at 160 °C, but if the values refer to fresh seeds, then there are no significant differences, as the extract obtained after treatment at 180 °C contained twice as many other substances such as sugars that do not contribute to the antioxidant activity. No significant differences were found with regard to the DPPH^•^ antiradical activity between samples.

The antioxidant capacity values determined as the capacity to scavenge certain free radicals are much higher than those obtained in previous studies using whole dates. For example, the TEAC values obtained in the present work by the ABTS^•^ method are well above values such as 0.67 mmol TE/g dried extract [2]. In comparison with other plant extracts such as strawberry, raspberry, orange, spinach, and broccoli, date seed extract has a 10–100 times higher antioxidant power [22]. In comparison with other extracts obtained from the seeds of different date varieties, such as the values reported by Radfair et al. [23] of between 16–21 mg/L or those also reported by Juhaimi et al. [9], the DPPH^•^ values obtained by applying a hydrothermal treatment are much higher. However, they are similar to fractions in terms of antioxidant activities (DPPH^•^ values of EC50 of 0.37–0.34 obtained from date seed vs. 0.08–3.5 obtained previously from whole date) or even higher activity (ABTS^•^ values of 3.28–6.61 mmol Trolox/g of dry extract obtained from date seed vs. 0.02–0.67 obtained previously from whole date) with other phenolic profile, obtained from an extract formed after application of the same hydrothermal process using whole dates [2]. The high antioxidant capacity values show the interest that these extracts can have in the food industry. This treatment allows obtaining a phenolic extract at a rate of 3 kg per ton of seed. This is quite a lot, since due to the high activity of these extracts, their use in food would allow them to be used at low concentrations at which commercial antioxidants are used (50–100 ppm) in the formulation of food; for example, 3 kg of extracts would serve to formulate and protect 30 tons of vegetable oil from oxidation, or for each ton of seed, 30 tons of oil or juice or other foods could be formulated. It should also be taken into account that treating one ton of seed generates 13–14 kg of free fermentable sugars and between 8 and 9 tons of solids rich in dietary fiber for the formulation of foods rich in antioxidant fibers as demonstrated in other recent work [14]. The use of all its components could justify the application of a hydrothermal pretreatment, for which further economic studies would be necessary.

## 5. Conclusions

The date seed is one of the main by-products of the date industry. There has been great interest in planning a comprehensive recovery of date seeds. This recovery is enhanced by the application of a steam thermal treatment, as it facilitates the extraction and obtaining of important fractions, such as oil or fiber, as has been shown in previous studies, and above all by the extraction of high-added-value components such as bioactive compounds with important biological properties. The present work shows that the liquid fraction obtained after the application of a direct steam treatment at two temperatures allows the solubilization of a high quantity of phenolic compounds that makes it possible to obtain an extract which is rich in phenols with high antioxidant activity. Undoubtedly, these results, together with those of previous works, support the valorization of date seeds in components that can improve the quality and functionality of new foods, in addition to helping the date industry. The amount of extract that can be obtained by this method is about 3 kg of extract per ton of seeds, which is a high value considering that the amount in which these extracts are used in feed is very low, about 50–200 ppm per final product.

Further studies will be necessary to identify the remaining components of the extract, which should be mainly oligosaccharides, since sugars are the main companions of phenols in the final extracts, and as they come from the elution with a high percentage of ethanol, these sugars could be oligosaccharides with phenolic residues or acid oligosaccharides such as pectin, which would increase their activity and importance.

## Figures and Tables

**Table 1:** The amounts of phenols and total sugars loaded and eluted from the adsorption chromatographic column to obtain a phenol-rich extract using the two liquid fractions obtained after the treatment of date seeds at 160 and 180 °C for half an hour. Values are mean ± SD.

Fraction	Eluent	Phenolics		Sugars		Volume
		mg/mL	mg	mg/mL	mg	mL
		Treatment at 160 °C, 60 min				
**LF-160**	-	1.28 ± 0.05	1284.0	12.19 ± 0.14	12,190	1000
**LFD-160**	-	0.07 ± 0.00	68.0	7.46 ± 0.06	7455	1000
**I-160**	Water	0.05 ± 0.00	26.5	4.16 ± 0.10	2080	500
**II-160**	Water	0.04 ± 0.00	19.5	0.20 ± 0.01	100	500
**III-160** **(Ext-160)**	Ethanol:water (8:2 (*v/v*))	2.95 ± 0.08	1475.0	12.20 ± 0.03	6100	500
**IV-160**	Ethanol:water (8:2 (*v/v*))	0.06 ± 0.00	30.0	0.35 ± 0.01	175	500
**V-160**	Ethanol	0.01 ± 0.00	4.5	0.13 ± 0.01	65	500
		Treatment at 180 °C, 60 min				
**LF-180**	-	1.44 ± 0.03	1439	17.80 ± 0.16	17,800	1000
**LFD-180**	-	0.08 ± 0.00	79.5	14.90 ± 0.18	1490	1000
**I-180**	Water	0.16 ± 0.00	77.4	16.40 ± 0.23	8200	500
**II-180**	Water	0.10 ± 0.01	48.3	5.01 ± 0.21	2505	500
**III-180** **(Ext-180)**	Ethanol:water (8:2 (*v/v*))	3.41 ± 0.07	1706.3	18.22 ± 0.27	9110	500
**IV-180**	Ethanol:water (8:2 (*v/v*))	0.10 ± 0.00	51.3	4.30 ± 0.13	2150	500
**V-180**	Ethanol	0.04 ± 0.00	19.0	0.19 ± 0.01	95	500

**Table 2:** Composition of acid phenols, flavonoids, sugar degradation products, phenols, and total sugars and antioxidant activity of date seed liquid fractions treated at 160 and 180 °C for 60 min and their respective extracts obtained chromatographically. Values are mean ± SD. Different letters indicate significantly different results (*p* < 0.05).

	Steam Treatment Concentration (mg/L)			
Compounds	LF-180	LF-160	Ext-180	Ext-160
Phenolic acids				
Gallic acid	79.19 ± 0.98 ^a^	85.20 ± 2.14 ^a^	95.00 ± 1.12 ^b^	97.87 ± 2.41 ^b^
Protocatechuic acid	62.11 ± 1.04 ^a^	45.20 ± 1.85 ^b^	177.48 ± 5.62 ^c^	82.70 ± 3.21 ^ad^
Vanillic acid	17.99 ± 0.41 ^a^	8.68 ± 0.25 ^b^	59.54 ± 4.63 ^c^	18.01 ± 0.57 ^a^
p-hydroxybenzoic acid	13.80 ± 0.91 ^a^	7.62 ± 0.26 ^b^	54.22 ± 2.17 ^c^	13.80 ± 1.22 ^a^
Syringic acid	0.20 ± 0.01 ^a^	Traces	3.98 ± 0.01 ^b^	Traces
**Flavonoids**				
Luteolin	6.66 ± 0.97 ^a^	10.35 ± 1.22 ^b^	62.29 ± 3.48 ^c^	8.44 ± 1.00 ^ab^
Quercetin	Traces	0.87 ± 0.02 ^a^	18.16 ± 2.41 ^b^	Traces
**Degradation products**				
Hydroxymethylfurfural	2868.6 ± 67.1 ^a^	118.4 ± 8.28 ^b^	5143.1 ± 87.0 ^c^	1024.0 ± 35.9 ^d^
Furfural	115.6 ± 22.4 ^a^	0.4 ± 0.0 ^b^	632.7 ± 18.0 ^c^	30.7 ± 2.1 ^d^
**Total compounds**				
Total phenolics	1438.7 ± 29.3 ^a^	1284.1 ± 45.2 ^a^	3412.7 ± 69.8 ^b^	2950.1 ± 74.6 ^c^
Total sugars	17,803.4 ± 159.8 ^a^	12,194.1 ± 140.1 ^a^	18,221.4 ± 273.0 ^c^	12,203.2 ± 33.0 ^c^
**Antioxidant activity**				
DPPH^•^-scavenging capacity EC_50_ (mg/L)			0.37 ± 0.01 ^a^	0.34 ± 0.00 ^a^
ABTS^•^-scavenging capacity				
(TEAC: mmol TE/g dried extract)			3.28 ± 0.09 ^a^	6.61 ± 0.15 ^b^
(TEAC: mmol TE/kg fresh seed)			40.46 ± 0.19 ^a^	41.01 ± 0.95 ^a^
ORAC-scavenging capacity				
(mmol TE/g dried extract)			9.91 ± 0.84 ^a^	12.82 ± 0.58 ^b^
(mmol TE/kg fresh seed)			121.84 ± 10.35 ^a^	79.49 ± 3.62 ^b^

## Data Availability

The data are contained within the article.
